# Explainable machine learning for risk stratification following TAVI

**DOI:** 10.1177/20552076261466380

**Published:** 2026-08-03

**Authors:** Md. Ahasan Atick Faisal, Ruba Sulaiman, Muhammad E. H. Chowdhury, Faycal Bensaali, Abdulrahman Alnabti, Huseyin Cagatay Yalcin

**Affiliations:** 1 Biomedical Research Center, QU Health, 61780 Qatar University, Doha, Qatar; 2 Department of Electrical Engineering, College of Engineering, 61780 Qatar University, Doha, Qatar; 3 Heart Hospital, 36977 Hamad Medical Corporation, Doha, Qatar; 4 Department of Biomedical Science, College of Health Sciences, QU Health, 61780 Qatar University, Doha, Qatar; 5 Department of Mechanical and Industrial Engineering, College of Engineering, 61780 Qatar University, Doha, Qatar

**Keywords:** transcatheter aortic valve implantation, machine learning, explainable AI, risk stratification, outcome prediction

## Abstract

**Objectives:**

Transcatheter Aortic Valve Implantation (TAVI) is a minimally invasive procedure for treating severe aortic stenosis, but accurately predicting post-operative outcomes remains a challenge. This study employs machine learning (ML) techniques to predict four critical outcomes following TAVI: 30-day complications, mortality, Conduction Abnormalities (CA), and Paravalvular Leak (PVL).

**Methods:**

Using a dataset of 256 patients from Hamad Medical Corporation Heart Hospital TAVI program, we processed 54 clinical parameters through several preprocessing methods such as multiple imputation for missing values and synthetic data generation using Synthetic Minority Oversampling Technique (SMOTE) to address missing data and class imbalance. Various machine learning models, including Support Vector Machines (SVM), K-Nearest Neighbors (KNN), and Artificial Neural Networks (ANN), were trained and evaluated.

**Results:**

The models demonstrated strong predictive performance across all tasks, with SVM and KNN models achieving the highest ROC-AUC scores. SHapley Additive exPlanations (SHAP) analysis revealed key predictors such as diabetes, pacemaker, dyslipidemia and type of valve, providing insights into the factors contributing to each prediction.

**Conclusion:**

Our findings suggest that ML, combined with explainability analysis, can effectively identify high-risk patients and potentially improve clinical decision-making in TAVI procedures.

## Introduction

Aortic stenosis (AS) is one of the most common valvular heart diseases, particularly in the aging population, and without timely intervention, it can lead to severe outcomes with high morbidity and mortality rates.^1^ Transcatheter Aortic Valve Implantation (TAVI) has become a groundbreaking alternative to Surgical Aortic Valve Replacement (SAVR) for patients with severe symptomatic AS, especially those at high or prohibitive surgical risk.^2,3^ Over the years, TAVI has gained widespread acceptance and has now expanded to include patients with intermediate and even low surgical risk, thanks to advancements in relevant technology and patient management.^4,5^

Despite the growing use of TAVI, the complexity of patient selection and outcome prediction remains a significant challenge. Accurate risk stratification is essential for determining which patients will benefit the most from TAVI, as these patients often present with multiple comorbidities and diverse clinical profiles.^6^ Traditional risk assessment models, such as the EuroSCORE and the Society of Thoracic Surgeons (STS) score, have been widely used to predict outcomes like 30-day mortality following cardiac procedures.^7,8^ However, these models were not specifically designed for TAVI, and their predictive power may be limited when applied to this unique patient population.^9–13^ In response, TAVI-specific risk models, such as the TAVI2-SCORE and the CoreValve model, have been developed but these still leave room for improvement.^14–17^

Recent advances in artificial intelligence, particularly Machine Learning (ML), have introduced powerful tools for improving outcome prediction in clinical settings. ML algorithms, capable of learning complex, nonlinear patterns in large datasets, have shown promise in various cardiovascular applications, including outcome prediction for TAVI patients.^18–21^ Unlike traditional statistical models, ML algorithms can account for a wider range of variables without requiring predefined assumptions about the data, making them highly adaptable and robust.^22^ However, most of the ML studies have targeted only one outcome variable like mortality while only few studies tried to predict more than one outcome.^23–27^ For more information regarding the application of ML for predicting the TAVI outcomes, we direct readers to our recent review article.^21^

While ML models have demonstrated impressive predictive capabilities, their widespread clinical adoption has been hindered by the ”black box” nature of many algorithms, which limits clinician trust and transparency in decision-making. Explainable Artificial Intelligence (XAI) has emerged as a critical framework to address this limitation by providing interpretable insights into model predictions. Recent advances in XAI have shown promise across diverse medical applications, including lung cancer detection,^28^ Internet of Medical Things (IoMT) systems,^29^ breast cancer classification,^30^ cardiac disease prediction,^31^ and diabetes diagnosis.^32,33^ Among XAI techniques, SHapley Additive exPlanations (SHAP) has gained particular prominence due to its model-agnostic nature and ability to provide both local (individual prediction) and global (overall model behavior) interpretability, making it especially valuable for clinical decision support systems.

TAVI program in Hamad Medical Corporation Heart Hospital (HMC-HH) is one of the leading TAVI programs in the region with excellent outcomes.^34^ In this study, we collaborated with HMC-HH TAVI program to leverage ML techniques to develop a predictive model for multiple TAVI outcomes, including 30-day complications, mortality, Conduction Abnormalities (CA), and Paravalvular Leak (PVL). Unlike most existing studies that focus on single outcomes (predominantly mortality), we simultaneously predict four distinct clinical outcomes, each requiring tailored feature selection and model optimization strategies. We deliberately employ classical ML algorithms rather than complex deep learning architectures to maintain model interpretability—a critical requirement for clinical adoption and regulatory acceptance. Furthermore, we apply comprehensive SHAP analysis across all four outcomes to provide clinically actionable insights into feature contributions, going beyond simple performance metrics to deliver interpretable decision support. By combining the predictive power of ML with model interpretability, we seek to provide a robust tool for enhancing patient selection and clinical decision-making in TAVI procedures.^35–37^

### Dataset description

The dataset used in this study was sourced from the HMC-HH in Doha, Qatar, and includes data from 256 patients who underwent TAVI between 2013 and 2023. The data was collected from the TAVI registry database, and relevant clinical parameters were extracted and organized into a tabular format for analysis.

The dataset used in this study consists of 54 clinical parameters, excluding the patient ID, which have been categorized into distinct groups for more structured analysis.

• **Demographics:** This category includes key patient characteristics such as age, gender, weight, height, and Body Mass Index (BMI).
• **Vitals:** Includes systolic and diastolic Blood Pressure (BP), as well as heart rate before and after the procedure.
• **Cardiac Factors:** Includes parameters directly related to cardiac health and previous interventions, such as Infective Endocarditis (IE), the presence of a pacemaker, Implantable Cardioverter Defibrillator (ICD), Coronary Artery Bypass Graft (CABG), Percutaneous Coronary Intervention (PCI), Atrial Fibrillation (AF), History of Valvular Disease (HVD), prior TAVI, and Heart Failure (HF).
• **Risk Factors:** This category captures pre-existing risk factors, including diabetes, hypertension, dyslipidemia, Chronic Kidney Disease (CKD), cancer, smoking status, Familial Hypercholesterolemia (FH), prior stroke, Transient Ischemic Attack (TIA), Carotid Stenosis greater than 50% (CS > 50%), and Peripheral Vascular Disease (PVD).
• **Basic Laboratory Values:** Key lab results such as platelet count, bilirubin, International Normalized Ratio (INR), creatinine, and hemoglobin are included in this category.
• **Echocardiographic Measures (Echo):** This category contains diagnostic echo parameters including peak and mean transvalvular aortic gradients, Aortic Valve Area (AVA), Left Ventricular Posterior Wall Dimension (LVPWd), Interventricular Septal Dimension (IVSd), Left Ventricular Ejection Fraction (LVEF%), abnormal Right Ventricular Function (RVF), abnormal Right Ventricular Size (RVS), Right Ventricular Systolic Pressure (RVSP), and Aortic Annulus Dimensions (AD, ADL, ADS).
• **Symptoms:** Patient symptoms at presentation include Shortness Of Breath (SOB), angina, syncope, abnormal Electrocardiogram (ECG), and Carotid Stenosis greater than 50% (CS > 50%).
• **Procedure-Related Variables:** Factors related to the TAVI procedure, such as the type of valve used (Medtronic Evolut (ME) vs. Edwards SAPIEN (ES)), access route to the Right Aortic (RA), and valve size.

Institutional Review Board (IRB) approval was granted by Hamad Medical Corporation (HMC) Medical Research Center (MRC). Heart Hospital is among the specialized hospitals functioning under HMC. All the included data is from deindentified retrospective TAVI patients and informed consent for participants was waived by the medical research committee. The data collection process fully adhered to the information governance policies of the Health Information Department at HMC. All study procedures and protocols were performed following the declaration for Helsinki.

## Data preprocessing

The raw data contained some missing values for few study parameters. Also, since the value of the parameters were not normalized, these was not readily available for training any ML algorithms. The data preprocessing steps involved several stages to ensure the quality and reliability of the dataset used for the analysis. These steps are discussed in the upcoming sections.

### Data cleaning

The initial dataset contained missing values across various parameters. Patients with more than 10 missing parameter values were removed from the dataset. Similarly, parameters with missing values in more than 10% of the patients were also excluded from the analysis. 3 patients were removed through this step. For the remaining missing values, we employed the Multiple Imputation by Chained Equations (MICE)^38^ technique to impute the missing values. To prevent data leakage, the MICE imputer was fitted on the training set only (after the train/test split), and the fitted imputer was then applied to both the training and test sets. This ensured that no information from the test set influenced the imputation parameters. Compared to simpler imputation methods such as mean, median, or constant value imputation, MICE provided superior results and maintained the integrity of the data.

### Data transformation

To prepare the data for ML algorithms, we normalized the data using both the Standard Scaler and Min-Max Scaler. The Standard Scaler normalized the data to have a mean of 0 and a standard deviation of 1, which is represented by the equation:

(1) $$z=\frac{\left(x-\mu \right)}{\sigma }$$

where *x* is the original value, *μ* is the mean, and *σ* is the standard deviation. The Min-Max Scaler scaled the data to a range of [0, 1] using the following equation:

(2) $$x^{\prime }=\frac{\left(x-x_{\text{min}}\right)}{\left(x_{\text{max}}-x_{\text{min}}\right)}$$

where *x*_min_ and *x*_max_ are the minimum and maximum values of the feature, respectively.

### Data splitting

The dataset was randomly split into training and test sets, with 80% of the data allocated to the training set and the remaining 20% to the test set. An additional 5-fold cross validation was performed withing the training data to tune the model hyperparameters.

### Data augmentation

One of the significant challenges in this study was the imbalanced nature of the dataset, where the majority of patients experienced positive outcomes following TAVI, and only a small proportion encountered negative outcomes. Specifically, for the 30-day complications, there were 198 positive and 40 negative cases; for mortality, 158 positive and 45 negative cases; for conduction abnormalities (CA), 179 positive and 62 negative cases; and for paravalvular leak (PVL), 216 positive and 22 negative cases in the clean dataset. Training ML models on such an imbalanced dataset often leads to a bias towards the majority class, resulting in poor predictive performance for the minority class (i.e., the negative outcomes).^39^

To mitigate the effects of this imbalance and enhance the model’s ability to predict negative outcomes, we employed data augmentation techniques to balance the training set. Specifically, we used the Synthetic Minority Over-sampling Technique (SMOTE)^40^ algorithm to generate additional synthetic examples of the minority class (negative outcomes). To prevent data leakage, SMOTE was applied strictly to the training set after the train/test split had been performed. The test set remained completely independent and was not involved in any synthetic data generation, ensuring that model evaluation was performed on unseen, original data only. SMOTE works by creating new instances of the minority class by interpolating between existing examples. This approach helps to increase the representation of negative cases in the training data, thereby ensuring that the models are trained on a more balanced dataset.

The SMOTE algorithm was applied separately to each of the outcome variables (30D complications, mortality, CA, and PVL) to equalize the number of positive and negative cases in the training set. As a result, the models could be trained without bias towards the positive class, ultimately improving their ability to predict negative outcomes effectively. This balancing of the dataset allowed for the development of more robust and generalizable machine learning models, as it ensured that the models were equally attentive to both positive and negative outcomes during training.

## Feature ranking and machine learning

This study began with a comprehensive set of 54 clinical features to develop ML models for predicting outcomes of TAVI. However, not all features contributed equally to model performance. To enhance model accuracy and interpretability, a systematic feature selection process was carried out.

Initially, a Pearson correlation analysis^41^ was performed to identify and remove highly correlated features with a correlation coefficient above 0.9. This step helped reduce multicollinearity and redundancy among predictors, ensuring the independence of retained features.

Following this, feature ranking algorithms available in MATLAB (Introduction to Feature Selection: https://www.mathworks.com) were applied to evaluate the importance of each feature. The algorithms included Minimum Redundancy Maximum Relevance (mRMR),^42^ Neighborhood Component Analysis (NCA),^43^ Chi-Square Feature Selection,^44^ and the ReliefF algorithm.^45^ Each method evaluated feature relevance from a different perspective, and the features were ranked accordingly. The top-ranked features demonstrating the highest relevance were retained for model training.

Subsequently, a variety of ML algorithms were employed using the MATLAB Classification Learner (Classification Learner: https://www.mathworks.com) tool to predict TAVI outcomes based on the selected features. These included traditional classifiers such as Decision Trees,^46^ Logistic Regression,^47^ and Discriminant Analysis,^48^ as well as more advanced models like Support Vector Machines (SVM),^49,50^ K-Nearest Neighbors (KNN),^51^ and Artificial Neural Networks (ANNs).^52–54^ Additionally, kernel approximation techniques,^55^ Naive Bayes classifiers,^46^ and ensemble methods such as Bagging,^56^ Boosting,^57^ and Random Forests^58^ were utilized to explore diverse modeling paradigms.

The performance of these models using the selected features is discussed in the following sections.

## Model training

The process of training ML models to predict the outcomes of TAVI, involved several interdependent tasks, each requiring careful consideration and iteration. The key tasks included the selection of feature ranking techniques, determining the optimal number of features, selecting the best model, and tuning the hyperparameters. Given the complexity of these tasks, a systematic and iterative approach was necessary to identify the best combination of features, models, and hyperparameters.

### Selection of feature ranking techniques

As discussed in the feature ranking section, several advanced feature ranking techniques were employed to rank the importance of features. These techniques included Minimum Redundancy Maximum Relevance (mRMR),^42^ Neighborhood Component Analysis (NCA),^43^ Chi-Square Feature Selection,^44^ and the ReliefF algorithm.^45^ The selection of the feature ranking technique was not a straightforward decision; instead, it required multiple rounds of experimentation. We evaluated the impact of each ranking technique on model performance by training models using the top-ranked features from each technique and selected the one with the best performance.

### Determining the number of features

The number of features used to train the model is crucial to balancing model complexity and predictive power. Too many features can lead to overfitting, while too few can result in underfitting.^59^ Therefore, we experimented with different numbers of top-ranked features from each ranking technique. The performance of the models was assessed using cross-validation to determine the optimal number of features that maximized accuracy while maintaining generalizability.

### Model selection

The next step was to select the best ML model from the various algorithms available in the MATLAB Classification Learner tool, including Decision Tree, Discriminant Analysis, Support Vector Machine (SVM), Logistic Regression, K-Nearest Neighbors (KNN), Kernel Approximation, Ensemble Methods, Artificial Neural Networks (ANN), and Naive Bayes. Each model was trained on the selected features, and its performance was evaluated using metrics such as accuracy, sensitivity, specificity, and area under the ROC curve (AUC-ROC).^60^ This process often required going back to earlier steps—such as re-ranking features or adjusting the number of features—to optimize the model’s performance.

### Hyperparameter tuning

After selecting the best-performing model, we focused on fine-tuning its hyperparameters to further enhance its predictive accuracy. Hyperparameters, which include parameters such as the number of neighbors (for KNN and Random Forest), regularization strength, kernel type (for SVM), and the number of neurons (for ANN), among others, play a critical role in model performance. To optimize these hyperparameters, we employed Bayesian Optimization,^61^ an exhaustive search method that systematically finds out the best set of hyperparameter values. Bayesian Optimization was applied using cross-validation to ensure that the selected hyperparameters not only maximized performance on the training set but also generalized well to unseen data.

### Iterative optimization

The process of model training was highly iterative, involving continuous back-and-forth between the selection of feature ranking techniques, the number of features, model selection, and hyperparameter tuning. Each iteration informed the next, allowing us to refine our approach progressively. For instance, initial results from model training could suggest the need to revisit feature selection or to explore alternative hyperparameter configurations. This iterative process was essential in identifying the best combination of features, models, and hyperparameters to achieve optimal predictive performance.

The outcome of this complex and iterative model training process was a finely-tuned ML model, optimized for predicting the outcomes of TAVI with high accuracy and robustness. The results of the final model and its performance metrics will be discussed in the subsequent sections.

## Results

In this section, we present the results of our analyses. We begin by discussing the findings from the statistical analysis, which highlights the correlation between input variables and the target outcomes. Following this, we explain feature importance, identifying the most significant input variables based on our feature analysis methods. Finally, we showcase the results obtained from the ML models.

### Statistical analysis

Our primary analysis cohort included patients who underwent implantation of a transcatheter valve at the HMC-HH. The dataset comprised 256 patients, whose clinical and demographic data were analyzed. We preformed statistical analysis to calculate the correlation between the input variables and the target variables (mortality, 30D complications, PVL, and CA). Categorical variables were compared using the Fisher exact test,^62^ while continuous variables were presented as mean ±standard deviation (SD) and compared using the Student t-test.^63^ For continuous variables, p-value reflects the likelihood that the means of the two groups are different due to random variation, while for categorical variables, it indicates the probability that the distribution of the categories differs between groups by chance alone. All statistical tests were two-sided with an alpha level of 0.05. Table 1 summarizes the p-values for each parameter for these outcomes. Statistically significant values are annotated with bold text and are underlined.

**Table 1.** Baseline characteristics and P-values for clinical parameters and outcomes.

​	Variables^*^	Mean ± STD	p (30D)	p (Mort)	p (PVL)	p (CA)
DG	Age	73.41 ± 8.57	0.461	0.861	0.798	0.867
	Male	51.68 %	0.543	**0.033**	0.519	0.844
	Weight	81.07 ± 18.07	0.859	0.461	0.205	0.623
	Height	143.43 ± 51.29	0.141	0.091	0.762	0.297
	BMI	31.07 ± 7.64	0.779	0.415	**0.033**	0.125
Vitals	Systolic BP	127.65 ± 21.47	0.529	0.790	0.726	0.081
	Diastolic BP	69.08 ± 11.23	0.145	0.564	0.473	0.225
	Heart Rate	70.70 ± 12.81	0.940	0.055	0.431	0.973
Cardiac Factors	IE	1.68 %	0.363	0.644	0.580	0.237
	Pacemaker	7.14 %	**0.036**	**0.006**	0.576	**0.003**
	ICD	0.84 %	0.522	0.448	0.652	0.432
	CABG	11.76 %	0.227	0.857	0.728	0.170
	PCI	31.09 %	**0.046**	0.161	0.686	0.531
	AF	28.15 %	0.891	**0.023**	0.960	0.995
	HVD	15.97 %	0.115	0.115	0.262	0.927
	Prior TAVI	7.98 %	**0.017**	0.255	0.560	0.788
	HF	32.35 %	0.067	**0.011**	0.258	0.451
Risk Factors	Diabetes	76.05 %	**0.019**	0.558	**0.000**	0.065
	Hypertension	89.50 %	0.657	0.818	0.159	0.564
	Dyslipidemia	53.36 %	0.596	0.586	0.437	0.753
	CKD	29.41 %	0.125	**0.009**	0.807	0.602
	Cancer	9.66 %	0.960	**0.018**	0.392	0.332
	Smoker	17.65 %	0.393	0.748	0.493	0.626
	FH	1.26 %	0.435	0.587	0.144	0.305
	Stroke	5.88 %	0.232	0.449	0.723	0.602
	TIA	2.10 %	0.308	0.902	0.403	0.077
	CS > 50%	2.10 %	0.295	0.448	0.419	0.617
	PVD	5.04 %	0.108	0.997	0.258	0.464
LAB	Platelet	216.17 ± 82.24	0.585	0.634	0.590	0.127
	Bilirubin	8.90 ± 7.47	0.663	0.468	0.447	0.659
	INR	1.15 ± 0.29	0.664	0.578	0.587	0.425
	Creatinine	118.88 ± 111.69	**0.017**	**0.033**	0.406	0.817
	Hemoglobin	11.51 ± 1.63	0.246	**0.039**	0.169	0.368
Echo	Peak Gradient	68.92 ± 22.30	0.609	0.124	0.151	0.263
	Mean Gradient	46.03 ± 37.94	**0.034**	0.380	0.534	0.379
	AVA	0.80 ± 0.22	0.255	0.412	0.720	0.099
	LVPWd	1.12 ± 0.21	0.974	0.805	0.783	0.946
	IVSd	1.19 ± 0.23	0.768	0.410	0.545	0.360
	LVEF(%)	51.37 ± 10.18	0.381	0.783	0.536	0.303
	Abnormal RVF	7.56 %	0.432	0.435	0.111	0.682
	Abnormal RVS	2.10 %	0.865	0.274	**0.015**	0.764
	RVSP	38.78 ± 13.88	0.213	0.208	0.737	0.703
	ADL	21.44 ± 3.20	0.543	0.361	0.387	0.584
	ADS	24.71 ± 3.56	0.277	0.130	0.864	0.561
	AD	23.01 ± 2.60	0.695	0.548	0.607	0.804
Symptoms	SOB	42.44 %	0.553	0.134	0.735	0.436
	Angina	7.14 %	0.458	0.421	0.306	0.960
	Syncopy	3.78 %	0.630	0.733	0.748	0.956
	CS > 50%	0.42 %	**0.026**	0.349	0.651	0.437
	Abnormal ECG	4.98 ± 6.49	0.390	0.749	0.578	0.578
Proc	Valve Type (ES/ME)	21.43%/78.57%	0.611	**0.043**	0.590	**0.014**
	RA Access	0.84 %	0.532	0.455	0.652	0.434
	Valve Size	26.80 ± 2.96	0.266	0.148	0.837	**0.026**

*Statistically significant values (p < 0.05) are presented in bold. DG = Demographics; Proc = Procedure; 30D = 30D Complications; Mort = Mortality; IE = Infective Endocarditis; ICD = Implantable Cardioverter Defibrillator; CABG = Coronary Artery Bypas Graft; PCI = Percutaneous Coronary Intervention; AF = Atrial Fibrillation; HVD = History of Valvular Disease; HF = Heart Failure; CKD = Chronic Kidney Disease; FH = Familial Hypercholesterolemia; TIA = Transient Ischemic Attack; CS = Carotid Stenosis; PVD = Peripheral Vascular Disease; INR = International Normalized Ratio; AVA = Aortic Valve Area; LVPWd = Left Ventricular Posterior Wall end diastole; IVSd = Interventricular Septal end diastole; LVEF = Left Ventricular Ejection Fraction; AAD = Aortic Annulus Diameter; RVF = Right Ventricle Function; RVS = Right Ventricle Size; RVSP = Right Ventricle Systolic Pressure; ADS = Annulus Diameter Short-Axis; ADL = Annulus Diameter Long-Axis; AD = Annulus Diamater; SOB = Shortness of Breath; BP = Blood Pressure; ES = Edwards SAPIEN; ME = Medtronic Evolut; RA = Right Aortic.

From the analysis, several clinical parameters were identified as significantly affecting different outcomes. For **30-day complications**, key factors include the presence of a **pacemaker** (p = 0.036), **percutaneous coronary intervention (PCI)** (p = 0.046), **prior TAVI** (p = 0.017), **diabetes** (p = 0.019), **creatinine levels** (p = 0.017), and **mean aortic valve gradient** (p = 0.034). These findings suggest that patients with these conditions or higher levels of creatinine and mean gradient are at a heightened risk for complications within 30 days of the procedure.

Regarding **mortality**, significant predictors include **gender** (male) (p = 0.033), the presence of a **pacemaker** (p = 0.006), **atrial fibrillation (AF)** (p = 0.023), **heart failure (HF)** (p = 0.011), **chronic kidney disease (CKD)** (p = 0.009), **cancer** (p = 0.018), **creatinine levels** (p = 0.033), **hemoglobin levels** (p = 0.039), **heart rate after the procedure** (p = 0.055), and the **valve type** (p = 0.043). These parameters indicate that male patients, those with pacemakers, AF, HF, CKD, cancer, and certain blood markers (e.g., creatinine and hemoglobin) have a higher risk of death after TAVI.

For **paravalvular leak (PVL)**, significant risk factors are **body mass index (BMI)** (p = 0.033), **diabetes** (p = 0.000), and **right ventricular size (RVS)** (p = 0.015). This suggests that higher BMI, the presence of diabetes, and abnormal RVS significantly increase the likelihood of developing PVL post-TAVI.

Lastly, **conduction abnormalities (CA)** were strongly associated with the presence of a **pacemaker** (p = 0.003), the **valve type** (p = 0.014) (the exact valve type is analyzed in a later section), and **valve size** (p = 0.026). These findings indicate that patients with pacemakers, those implanted with certain valve types, and sizes are more prone to experiencing conduction issues after the procedure.

### Feature importance

**Figure 1.**
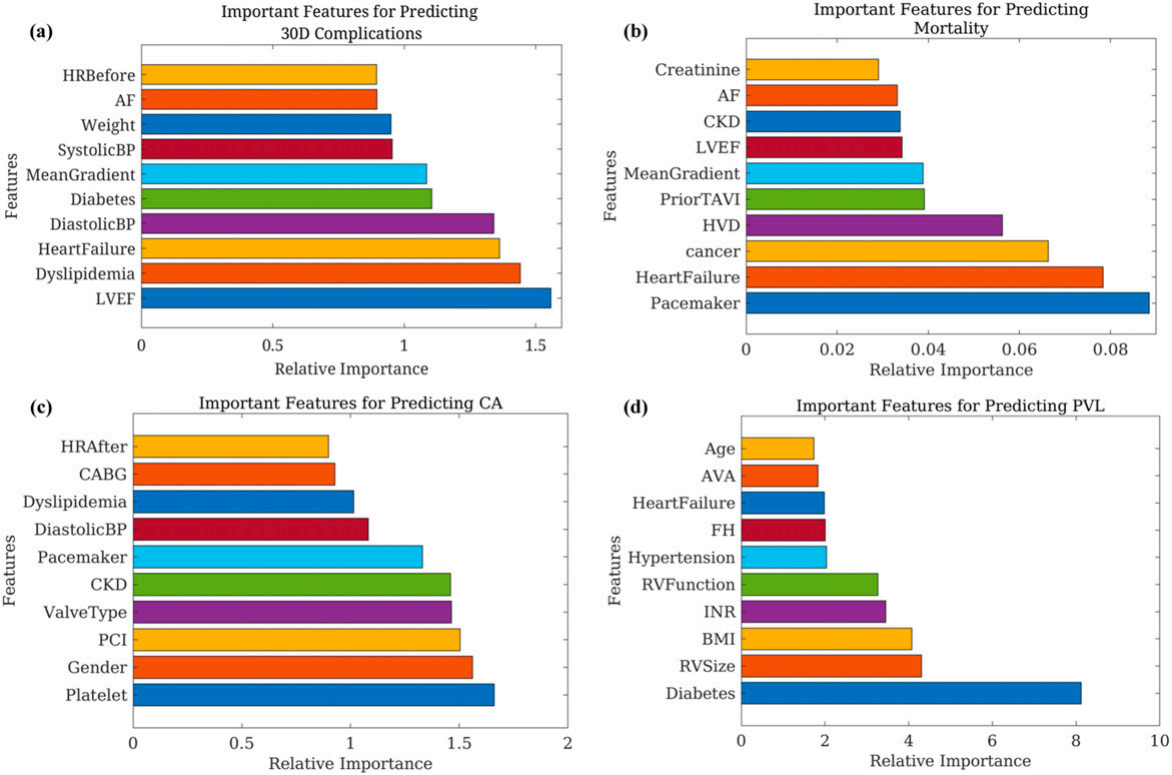
Top 10 features for (a) 30D Complication, (b) Mortality, (c) CA, and (d) PVL prediction.

To determine the most critical features for each task, we evaluated the performance of models trained using features selected by these different ranking techniques discussed earlier. The method that provided the best model performance for each task was chosen to rank the features, and the most important features identified through this process are depicted in Figure 1.

• **30D Complications:** Neighborhood Component Analysis (NCA) was selected as it provided the best performance for this task. As shown in Figure 1(a), the top features included Left Ventricular Ejection Fraction (LVEF), Dyslipidemia, Heart Failure, Diabetes, and Mean Gradient.
• **Mortality:** The ReliefF algorithm was found to be the most effective technique. Figure 1(b) highlights the most important features for predicting mortality, including Pacemaker presence, Heart Failure, Cancer, and Chronic Kidney Disease (CKD).
• **Conduction Abnormalities (CA):** Similar to 30D Complications, NCA was also selected for predicting CA. The most influential features, as seen in Figure 1(c), included Platelet count, Gender, Percutaneous Coronary Intervention (PCI), Valve Type, and Chronic Kidney Disease (CKD).
• **Paravalvular Leak (PVL):** The Chi-Square feature selection method was chosen for PVL prediction. Figure 1(d) displays the top features for this task, with Diabetes, Right Ventricular Size (RV Size), Body Mass Index (BMI), and Right Ventricular Function (RV Function) emerging as the most important predictors.

### Machine learning

The performance of various ML models was evaluated across four key outcomes of TAVI: 30D complications, mortality, CA, and PVL. The primary metric used to assess and select the best model was the Area Under the Receiver Operating Characteristic Curve (ROC-AUC), as it provides a comprehensive measure of the model’s ability to discriminate between positive and negative outcomes. Additionally, Precision, Recall, F1-Score, and Accuracy were considered to provide a more detailed understanding of model performance. The quantitative results from our experiments are summarized in Table 2 where the best performing models in terms of ROC-AUC score are annotated bold.

**Table 2.** Experimental ML Results for TAVI outcome prediction.

​	Model	Precision	Recall	F1-score	Accuracy	ROC-AUC
30D Complications	Decision Tree	89.19	91.67	90.41	84.09	0.78
	Logistic Regression	76.32	93.55	84.06	75.56	0.76
	Kernel Approximation	75.68	96.55	84.85	77.27	0.82
	Discriminant Analysis	75.68	93.33	83.58	75	0.76
	Naive Bayes	83.78	91.18	87.32	79.55	0.76
	**SVM**	**78.38**	**96.67**	**86.57**	**79.55**	**0.94**
	KNN	81.08	90.91	85.71	77.27	0.69
	Ensemble	89.19	84.62	86.84	77.27	0.72
	ANN	89.19	91.67	90.41	84.09	0.69
Mortality	Decision Tree	72.41	87.5	79.25	70.27	0.7
	Logistic Regression	58.62	89.47	70.83	62.16	0.78
	Kernel Approximation	65.52	83.36	74.51	64.68	0.72
	Discriminant Analysis	79.31	85.19	82.14	72.9	0.75
	Naive Bayes	58.62	85	69.39	59.46	0.75
	**SVM**	**100**	**85.29**	**92.06**	**86.49**	**0.81**
	KNN	86.21	92.59	89.29	83.78	0.81
	Ensemble	96.55	90.32	93.33	89.19	0.75
	ANN	75.86	91.67	83.02	75.68	0.78
CA	Decision Tree	75.76	75.53	74.63	62.22	0.53
	Logistic Regression	54.55	85.71	66.67	60	0.7
	Kernel Approximation	81.82	81.82	81.82	73.33	0.7
	Discriminant Analysis	54.55	85.71	66.67	60	0.7
	Naive Bayes	81.82	77.14	79.41	68.89	0.57
	SVM	100	75	85.71	75.56	0.76
	**KNN**	**72.73**	**96**	**82.76**	**77.78**	**0.83**
	Ensemble	90.91	81.08	85.71	77.78	0.7
	ANN	72.73	85.71	78.69	71.11	0.68
PVL	Decision Tree	80.49	94.29	86.84	77.78	0.66
	Logistic Regression	78.05	96.97	86.49	77.78	0.69
	Kernel Approximation	63.41	96.3	76.47	64.44	0.73
	Discriminant Analysis	80.49	94.29	86.84	77.78	0.62
	Naive Bayes	80.49	94.29	86.84	77.78	0.56
	SVM	80.49	97.06	88	80	0.87
	**KNN**	**82.93**	**100**	**90.67**	**84.44**	**0.95**
	Ensemble	78.05	96.97	86.49	77.78	0.7
	ANN	85.37	94.59	89.74	82.22	0.73

#### 30D complications

For predicting 30-day complications, the SVM model achieved the highest ROC-AUC score of 0.94, making it the best-performing model for this task. It also maintained a solid F1-Score of 86.57% and an accuracy of 79.55%. The ANN and Decision Tree models also performed well, both achieving an F1-Score of 90.41% and an accuracy of 84.09%, though with slightly lower ROC-AUC scores (0.69 and 0.78, respectively).

#### Mortality

In the mortality prediction task, the SVM model again demonstrated excellent performance with a ROC-AUC score of 0.81 and an F1-Score of 92.06%, along with an accuracy of 86.49%. The KNN model followed closely with a ROC-AUC of 0.81, an F1-Score of 89.29%, and an accuracy of 83.78%. The Ensemble method also showed strong results with an F1-Score of 93.33% and a ROC-AUC score of 0.75.

#### Conduction abnormalities (CA)

For CA, the KNN model achieved the highest ROC-AUC score of 0.83, making it the top performer for this task. It also had an F1-Score of 82.76% and an accuracy of 77.78%. The SVM model showed robust performance with a ROC-AUC score of 0.76 and an F1-Score of 85.71%, supported by an accuracy of 75.56%. The Ensemble method also performed well, achieving an F1-Score of 85.71% and an accuracy of 77.78%, with a ROC-AUC of 0.7.

#### Paravalvular leak (PVL)

In predicting PVL, the KNN model excelled with the highest ROC-AUC score of 0.95, along with an F1-Score of 90.67% and an accuracy of 84.44%. The SVM model also demonstrated strong performance with a ROC-AUC score of 0.87 and an F1-Score of 88%, achieving an accuracy of 80%. The Artificial Neural Network (ANN) model performed well, with a ROC-AUC score of 0.73, an F1-Score of 89.74%, and an accuracy of 82.22%.

Across all four tasks, the SVM and KNN models consistently demonstrated the highest ROC-AUC scores, indicating their superior ability to discriminate between positive and negative outcomes. The KNN model, in particular, performed exceptionally well in predicting conduction abnormalities and paravalvular leak, while the SVM model excelled in predicting 30D complications and mortality.

### Visualization of results

To provide a comprehensive understanding of the model performance, we have visualized the results using confusion matrices and Receiver Operating Characteristic (ROC) curves for each of the four outcome prediction tasks: 30D complications, mortality, CA, and PVL.

**Figure 2.**
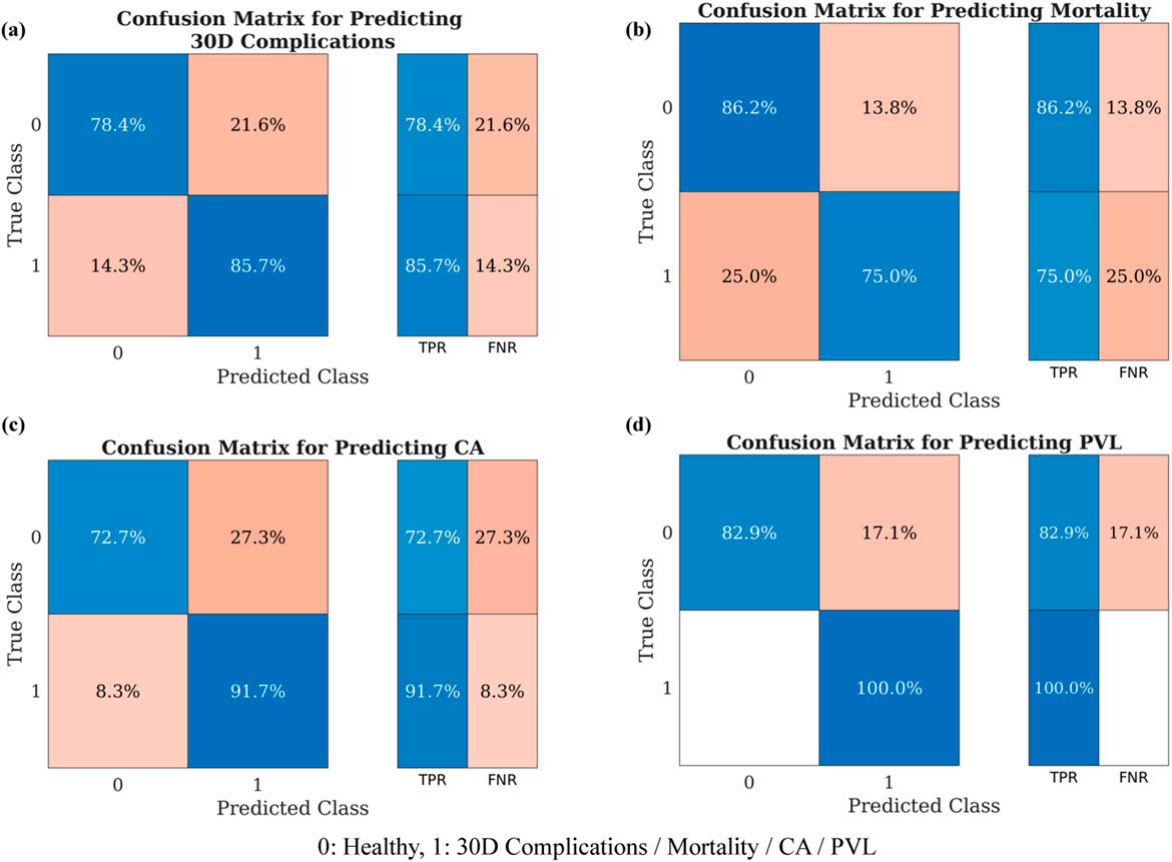
Confusion matrices for (a) 30D Complication, (b) Mortality, (c) CA, and (d) PVL prediction.

The confusion matrices presented in Figure 2 (panels a-d) depict the performance of the best models in predicting the outcomes of TAVI. Each matrix shows the distribution of true positive (TP), true negative (TN), false positive (FP), and false negative (FN) predictions, providing a clear understanding of the model’s accuracy and error distribution. The True Positive Rate (TPR) and False Negative Rate (FNR) are also depicted alongside each confusion matrix, offering additional metrics to assess the balance between sensitivity and specificity.

**Figure 3.**
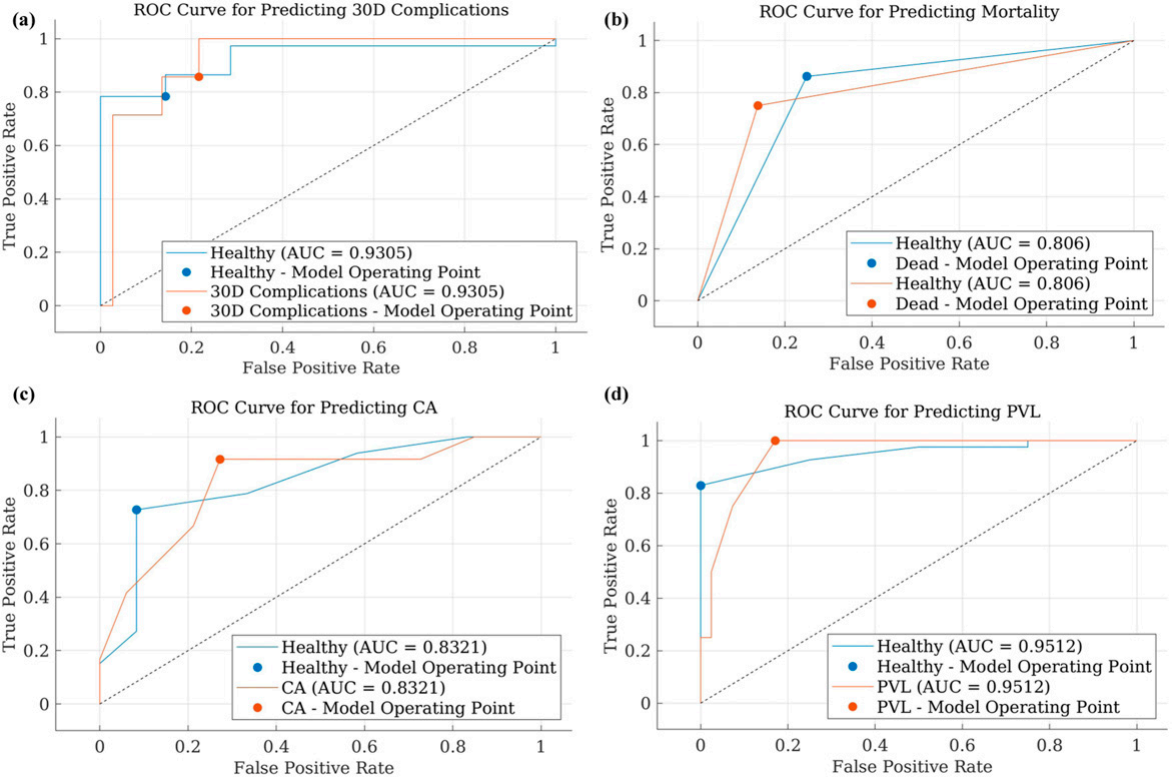
ROC curves for (a) 30D Complication, (b) Mortality, (c) CA, and (d) PVL prediction.

Figure 3 (panels a-d) displays the ROC curves for each outcome prediction task, which plot the True Positive Rate (sensitivity) against the False Positive Rate (1-specificity) at various threshold settings. The ROC curves provide a graphical representation of the model’s ability to distinguish between positive and negative cases across different decision thresholds. The Area Under the Curve (AUC) is also provided, quantifying the overall performance of the model in terms of its discriminatory power.

These visualizations shows the effectiveness of the selected models in predicting various TAVI outcomes and highlight the models’ strengths and potential areas for improvement.

## Discussion

### Model explanation

**Figure 4.**
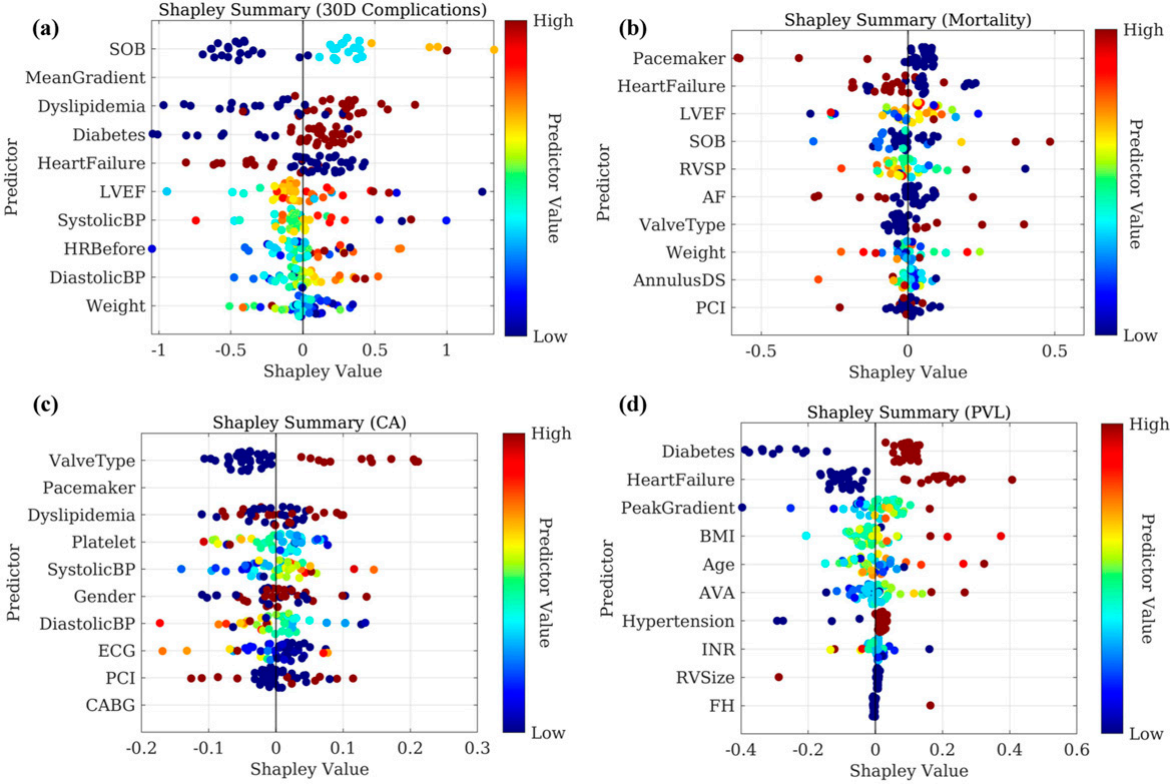
Shapley explanation for (a) 30D Complication, (b) Mortality, (c) CA, and (d) PVL prediction.

To further understand the decision-making process of the best-performing models, we applied the Shapley value algorithm to interpret the predictions made on the test dataset. Shapley values, derived from cooperative game theory, provide a measure of each feature’s contribution to the prediction, offering insights into the model’s reasoning.^64,65^ The Shapley summary plots for each outcome prediction task - 30D complications, mortality, CA, and PVL - are shown in Figure 4.

#### 30D complications

As shown in Figure 4(a), several features were identified as key contributors to predicting 30-day complications. Features such as shortness of breath (SOB), mean gradient, dyslipidemia, diabetes, heart failure, and left ventricular ejection fraction (LVEF) were among the most influential.

**Shortness of Breath (SOB):** SOB was highlighted as a significant predictor. This aligns with existing literature where SOB is often linked to poorer outcomes in TAVI patients^20^ due to its association with heart failure.^66^

**Mean Gradient:** A higher mean gradient across the aortic valve was positively associated with improved survival rates, consistent with studies that suggest patients with low-gradient aortic stenosis have a more advanced disease state and worse outcomes post-TAVI.^67–70^

**Dyslipidemia and Diabetes:** Both metabolic disorders were significant contributors, echoing their established roles as risk factors for cardiovascular diseases, including TAVI outcomes.^71,72^

#### Mortality

Figure 4(b) illustrates the most important predictors for mortality, including pacemaker presence, heart failure, LVEF, and SOB.

**Pacemaker Presence:** The model indicated pacemaker presence as a strong predictor of mortality, which could be related to the association between conduction system disease and worse survival outcomes in TAVI patients.^73,74^

**Heart Failure and LVEF:** Both heart failure and reduced LVEF were crucial predictors, underscoring the well-documented negative impact of left ventricular dysfunction on survival following TAVI, as supported by multiple studies.^25,26,75^

**Renal Function (Creatinine):** The significance of serum creatinine highlights the critical role of renal function, corroborating findings from the literature that link creatinine and chronic kidney disease with higher mortality in TAVI patients.^18,23,24,27,72,76^

#### Conduction abnormalities (CA)

For predicting conduction abnormalities, Figure 4(c) identifies valve type, pacemaker, and platelet count as key predictors.

**Valve Type and Pacemaker:** Interestingly, Medtronic Evolut significantly afftected the outcome of CA prediction. The patients with Medtronic Evolut valve had higher chances of developing CA compared to the patients equipped with Edwards SAPIEN valve.

**Pacemaker:** This finding are consistent with studies indicating that certain valve types and pre-existing conduction disturbances increase the likelihood of post-TAVI conduction abnormalities, necessitating pacemaker implantation.^77^

**Platelet Count:** The significance of platelet count as a predictor could reflect its role in perioperative bleeding risk, which is associated with a higher incidence of conduction abnormalities.^18,24^

#### Paravalvular leak (PVL)

As shown in Figure 4(d), diabetes, heart failure, and peak gradient were the most influential features for predicting PVL.

**Diabetes:** Interestingly, diabetes was the top predictor, which could relate to its association with systemic inflammation and adverse remodeling, increasing the risk of PVL.^69,72^

**Heart Failure and AV Gradient:** The influence of heart failure and AV gradient on PVL prediction is well-supported by literature, with low AV gradient often indicating more advanced aortic stenosis and worse hemodynamic outcomes.^6,67,68,70^

These Shapley value analyses provide valuable insights into the factors driving the models’ predictions, reflecting both established clinical knowledge and new potential areas of exploration in TAVI outcome prediction.

### Comparison with existing literature

**Figure 5.**
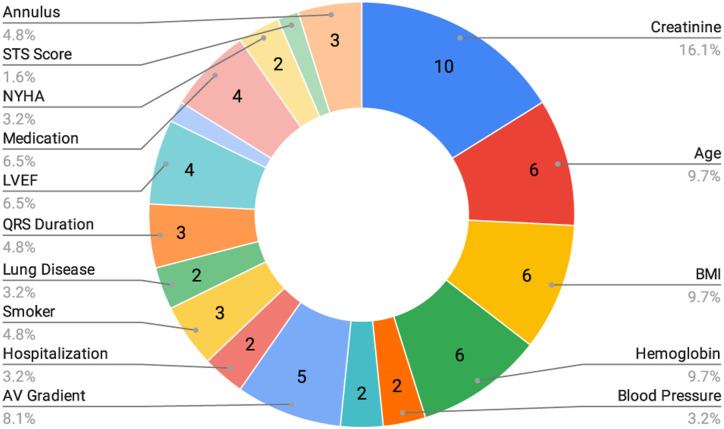
Top predictors of TAVI outcome found from 17 similar articles. The numbers in the chart indicates how many articles mentioned that predictor a important factor for TAVI outcome prediction.

Table 3 summarizes similar works on ML-based TAVI outcome prediction. It can be seen from the table that most of the works focus on mortality prediction only while in this work we explored 4 types of outcomes from the same database. This will provide better insights about how the different outcomes relate to each other. While our study results are promising and align well with those in the literature, it is important to note that the datasets used in previous studies differ significantly from ours, both in size and demographic composition, which means direct comparisons are not ideal. Therefore, instead of comparing the quantitative matrices, we will focus more on the top predictors that were discovered from our analysis and compare them with the existing literature. Recently, we systematically reviewed the literature to assess ML works applied to TAVI for outcome prediction.^21^ In that work, we identified the top predictors of TAVI outcome prediction from the ML-based papers. These predictors are also summarized here in Figure 5. In the figure, the numbers in the different colored pie slices, indicate the number of papers where that particular variable was mentioned as a key predictor.

**Table 3.** Summary of the existing literature on ML-based TAVI outcome prediction.

Ref	Predicted variable	Number of patients	Neg. Cases (%)	No. of predictors	Models	AUCROC
23	Mortality	1055	14.02	287	GBM	0.72
71	Mortality	54739	2.08	40+	LR, LightGBM, GBC, LDA, CatBoost	0.817
78	CA	151	-	8	8 Classical ML Models	0.84
76	Mortality	451	5.9	83	ANN, RF, SVM	0.96
24	Mortality	1428	11.5	44	XGBoost	0.82
68	Mortality	34840	3.3	155	Random Forest	0.75-0.79
75	Mortality	186	30D: 3.9 1Y: 13.5	42	Decision Tree	30D: 0.83, 1Y: 0.71
79	Mortality	1478	9.8	-	GTB, SVM, MLP, RFC, LR	0.70
25	Mortality	1791	10.5	16	RF, XGB, CATBoost, ANN	0.66
69	Mortality	270	11.1	115	Gradient Boosting Decision Tree (GBDT)	0.83
26	Mortality	1931	10.2	16	LR, RF, CatBoost	0.66
18	Bleeding Risk	Train: 5185, Val: 5043	-	100+	RF, NB, LR	0.78
27	Mortality	471	45	83	LR, RF, XGBoost, ANN	0.79
25	Mortality	11291	3.36	17	LR, XGBoost	0.66
72	Mortality	629	10.2	134	MLP, SVM, KNN, RF, NB	0.82

When comparing the predictors identified by our models with those frequently cited in the literature, several consistencies and novel findings emerge. Key predictors such as serum creatinine, age, BMI, and left ventricular ejection fraction (LVEF) were prominently featured both in our analysis and the literature, underscoring their critical role in predicting mortality and other adverse outcomes following TAVI. Creatinine, which reflects renal function, is the most frequently mentioned predictor in the literature and was also a significant predictor in our mortality and overall outcome models. Similarly, age and BMI, often associated with varying TAVI outcomes, were influential in both our models and the literature.

#### Valve type as a predictor of conduction abnormalities

In our study, the type of valve used in TAVI emerged as a significant predictor of CA. Out of the patients analyzed, 54 received an Edwards SAPIEN balloon-expandable valve and 202 patients received self-expandable Medtronic Evolut valve. Regarding conduction abnormalities, 62 patients developed conduction abnormalities post-TAVI. Among the patients who were equipped with the Edwards SAPIEN (ES) valve, 12.96% developed CA and among the patients who were equipped with the Medtronic Evolut (ME) valve, 29.41% developed CA. Statistical analysis revealed a p-value of 0.014, indicating a statistically significant association between the type of valve and the occurrence of conduction abnormalities. Medtronic Evolut valve has been included to the program from the beginning, whereas Edwards SAPIEN has been included more recently. The differences in outcomes may be due to the late inclusion of the SAPIEN valve to the program when it has been more successful.

Moreover, our SHAP analysis, which interprets the predictions of our best-performing ML model for CA, identified valve type as the top contributing feature for predicting conduction abnormalities. This suggests that valve type, specifically the Medtronic Evolut valve, plays a critical role in the risk of developing conduction abnormalities post-TAVI.

These findings highlight the importance of considering valve type during pre-procedural risk assessment for TAVI patients. While previous studies have identified other factors such as pacemaker status and pre-existing conduction disturbances, the strong association between the valve type and CA in our study provides new insight into valve selection as a potential risk factor. This observation warrants further investigation and validation in larger, more diverse cohorts to determine the underlying mechanisms and to refine risk stratification strategies for TAVI patients.

### Limitations

This study has several limitations that could affect the generalizability and robustness of the findings. The dataset comprises 256 patients collected over 10 years (2013-2023) from a single center at Hamad Medical Corporation, representing the real-world challenges of medical data acquisition in specialized procedures like TAVI. Some outcome events had relatively low occurrence rates (PVL: 22 cases, CA: 62 cases), which, combined with the initial 54 features, resulted in a lower events-per-variable ratio. To mitigate overfitting risks, we implemented rigorous data leakage prevention protocols (SMOTE applied only to training folds, MICE imputation fitted only on training data) and employed feature selection techniques to reduce dimensionality. While SMOTE helped address class imbalance, synthetic data generation may not fully capture the complexity of real-world cases, potentially introducing biases. The single-center nature of the data limits the model’s applicability across different populations and healthcare settings. Furthermore, the temporal span of the dataset introduces variability due to changes in clinical practices over the years, which might impact the relevance of the findings to current and future patients. Nevertheless, our key findings demonstrate consistency with existing literature and have been corroborated by clinical observations from the TAVI program at Hamad Medical Corporation, particularly regarding valve type effects on conduction abnormalities, lending credibility to the model’s clinical relevance. Another limitation is the absence of comparison with established clinical risk scores such as the STS Score or EuroSCORE II. These scores were not available in the TAVI registry database, as they are primarily designed to predict surgical mortality risk rather than the specific outcomes examined in this study (paravalvular leak and conduction abnormalities). Future studies incorporating these benchmark scores would provide valuable context for assessing the clinical utility of ML-based predictions. Regarding PVL prediction, the model achieved 100% sensitivity (all actual PVL cases were correctly identified) but 82.9% specificity (17.1% false positive rate). While the high sensitivity is clinically desirable to avoid missing PVL cases, the presence of false positives indicates the model is conservative in its predictions. Given the small number of original PVL cases (22 patients), external validation on larger, independent datasets is essential to confirm the model’s generalizability and reduce false positive rates.

## Conclusion

In this study, we applied ML techniques to predict key outcomes for patients undergoing TAVI. By leveraging a comprehensive dataset of 256 patients, we identified significant clinical parameters that affect 30-day complications, mortality, PVL, and CA. The use of advanced data preprocessing, feature engineering, and model interpretation techniques such as SHapley Additive exPlanations (SHAP) provided valuable insights into the most critical predictors for each outcome. Our findings indicate that parameters such as the presence of pacemakers, diabetes, creatinine levels, and valve type play a crucial role in predicting these outcomes. The application of ML allowed us to identify nonlinear interactions between multiple factors, which are often difficult to capture using traditional statistical models. Moreover, the interpretability of the models via SHAP values enables clinicians to better understand the underlying reasons for specific predictions, thus facilitating more informed decision-making. While the results are promising, further validation is required to generalize the models across diverse populations and clinical settings. Future work should also explore the integration of peri-procedural data and longer-term outcomes to enhance the robustness of the predictions. Nevertheless, this study demonstrates the potential of machine learning to significantly improve outcome prediction in TAVI patients, paving the way for more personalized and accurate risk stratification in clinical practice.

## Data Availability

The data used in this study can be made available upon a reasonable request to the corresponding author.*
